# Hospital Progress and Finance

**Published:** 1923-09

**Authors:** 


### HOSPITAL PROGRESS AND FINANCE.

### CONTRIBUTION SCHEMES WITHOUT COLLECTORS.

THE contribution scheme associated with the Royal Berkshire Hospital is making steady progress, and the experience of the agricultural and often scattered villages in the method of weekly collection is of interest, because it is often difficult to organise this properly since the residents, whether contributors or not, are busy people who can hardly spare the time to travel weekly the considerable distances involved. To overcome this obstacle one exceptionally scattered parish has evolved a satisfactory plan. By the kind co-operation of the Vicar, the clubroom of the village is lent each Monday to the scheme, when the local treasurer or one of the committee attends to receive the contributions. This has proved a great convenience to the contributors, who send or bring their money, and as often as not pay for several weeks on the occasion of each visit. This plan reduces the number of attendances, and saves time and trouble to the treasurer and to the subscribers alike. The latter choose their own week to make their payments; the former is saved the business of recruiting collectors, to whom, as has been explained, the duties would prove exacting in energy and time. Kintbury, as this village is called, seems, with the vicar's help, to have devised a plan which might commend itself to other villages where the parishioners are scattered far apart.

---

### PAY HOSPITALS SINCE THE WAR.

IT is now nine years since St. Chads Hospital, Birmingham, for paying patients, was established, and its record is in one sense an index of the ups and downs in the hospital situation during this time. It possesses a hundred beds, and the average number at first occupied rose steadily from 13.68 to 46.62, the highest figure yet attained, in 1919-20. In 1921 there was a sharp drop again to 29, and up to March last the average was still declining. Whereas 1,084 patients had been admitted in 1919, the number fell to 752 last year. This is attributed to the industrial depression, which has made the relatively moderate fees charged beyond the means of many previously able to afford them. Most of the patients, according to the little report printed by the committee, pay an inclusive fee for hospital accommodation and treatment, which is said to be much lower than the cost of other nursing homes. Anyone can apply for admission to the House-Governor, who acts as an almoner and arranges the amount of the inclusive fee, which is never more than twelve guineas a week. St. Chads gives to its nurses a certificate after three years' training, which includes systematic lectures from a sister-tutor and the resident medical officer. In short, it endeavours to meet the conditions defined by the advisory committee to the Ministry of Health for pay-hospitals, and in these pages the progress of the work has been watched with interest from the start. Indeed, the plan of a fee to include the services of the physician or surgeon was a step forward, and as the staff consists of hospital consultants a bridge was built where it was most needed, and continues to excite inquiries from distant places at home and abroad. If the voluntary hospital has to close beds for lack of means, the pay hospital may not be able to fill them for a kindred reason. The prospects, however, for both are likely to improve unless the international situation deprives Europe of all recovery.

---

### WIDER SUPPORT AND WAITING LISTS.

DOES a contributory or "insurance" scheme necessarily increase not only a hospital's income but the number of cases seeking admission? This has been, in the words of its Chairman, Mr. S. H. Burton, the undoubted experience of the Norfolk and Norwich Hospital. The contributory scheme there has excited great interest in more senses than one, and the question of extension is now pressing, though the year began with an overdraft exceeding £9,000. There are now some 70,000 workers who subscribe to the contributory scheme, and their generosity, together with the growing waiting list, makes the acceptance of new responsibilities unavoidable. So far as we know, however, this is the first formal admission that a successful contribution scheme, in recent times, is immediately accompanied by a larger list of cases waiting for admission and therefore raises problems as well as cash.

---

### AN APPEAL THAT FAILED.

THE effort to raise £20,000 for the Royal Northern Hospital by July 31 was a failure. Although the appeal was extensive, only £1,500 was received toward the fund. Consequently the board has no alternative except to close 70 beds, which means the refusal of 1,000 cases. By driving this home the board hopes to convert a failure into a success, and it is true that the autumn, after the holidays, is the harvest season of charity, while the appeal was necessarily issued at the less favourable moment of three months earlier in the year. The failure is the more disappointing because the hospital has shown much enterprise of late, including a form of "insurance" contribution from which a good deal was hoped and something was gained.

---

### THE NEW BRITISH CHARITIES ASSOCIATION.

UNDER the presidency of Lord Knutsford this Association has been formed to start, and to supervise, schemes for collecting money in aid of hospitals and other charities. The Association, being registered as a company limited by guarantee, cannot make profits for itself. The first scheme that it takes in hand will give a clue to its methods. This is for a competition, of the class lately made familiar in the different "Ballots." A sum of £30,000 will be given in prizes, which have been guaranteed by a familiar firm that is also helping with the large amount of advertisement necessary. Every subscriber of not less than 5s. will receive an entrance ticket for the competition, in which the first prize will be £12,000. While the Association will be the central collecting agency, it will supply free tickets to hospitals which are able to collect for themselves. The total proceeds will be divided among the collecting hospitals and existing distributive agencies. Thus the hospitals will have no expenses. On the face of it the Association promises to fulfil a need. The law in regard to ballots and competitions is capricious, and probably only an established agency can be sure of being always on the right side. These competitions have had a vogue that does not seem to be diminishing, but this makes it all the more desirable that they should not be exploited by improper hands.

### HOSPITAL SWEEPSTAKES IN IRELAND.

THE special committee on the Public Charitable Hospitals (Temporary Provisions) Bill, 1923, has been presented to the Dail. The committee, which was appointed in June, held nine meetings, and decided to take evidence upon the financial needs of the hospitals, and on the proportion which the necessary expenses of a sweepstake bear to the receipts. The committee agreed that the needs of the hospitals were undoubted, that the cost of maintenance is heavy, and that debts are great. It also found that 40 per cent. was the average proportion of necessary expenses to the receipts of a sweepstake. The expenses mainly consist of prizes, postage, rent, wages, advertising and printing. Apparently many sweepstakes suffered from the interception of large numbers of letters which the English postal authorities stopped. Sweepstakes, like most other satisfactions of human instinct, require to be controlled rather than forbidden, and, provided the good faith of the organisers be unquestionable and that they are amenable to reasonable supervision, the sweepstake may contribute, no less than the swings and the hoop-la, to the cause of charity.

### RESEARCH AT GREAT ORMOND STREET.

A RESEARCH department for the study of children's diseases is being formed at the Hospital for Sick Children, Great Ormond Street. The clinical material offered by the wards is to be available for laboratory study. A bio-chemist is to be appointed, so that the behaviour of living tissue may be studied by laboratory tests, and by these means it is hoped to add much to our knowledge, to improve methods of treatment, and to devise preventive measures. Such a department has been long desired, but the cost of the first year's working is being found by the Prudential Assurance Company, which is furnishing a sum of £2,000 for the new work.

### INSTITUTIONAL ADVERTISING AND ITS LIMITS.

IN a recent number of the "Fortnightly Review" there appeared an interesting plea by Mr. L. G. Brock for a central Charities Board, by which he meant a body to achieve for charities in general that which King Edward's Hospital Fund achieves for hospitals. It is pleasant to see their due given to the virtues of the voluntary system, and to the value of the uniform method of accounts; but while the hospitals are held up as an example of voluntary organization to which the mass of charities other than hospitals would do well to conform if they are to rid themselves of abuses, some of the criticisms levelled at them may also be studied with profit by hospital managers. For example, organised advertising on American methods is not unknown in the hospital world, and the writer puts his finger on its dangers, dangers which hospital men no less than others would do well to bear in mind:—

> "The professional 'organizing expert,' a recent and undesirable feature of charitable work, aims frankly at 'boosting' his charity as if it were a new soap or liver pill. Any expenditure is held to be justified if the net yield is substantial. But there is no real parallel between commerce and charity organization. From the commercial point of view it is no doubt perfectly sound to spend sixpence in order to sell three pennyworth of ice-cream for half a crown; but a charity which spends ten shillings to raise a pound is definitely harmful, because it tends to divert funds from other charities, which in their turn may be tempted to resort to equally or even more sensational methods. If the public knew the amount spent in advertising certain appeals in recent years, subscriptions to them would stop at once. For the moment the financial depression has checked this reckless advertising, but it will probably recur as soon as trade improves, unless some effective means can be devised to discourage it."

The competition for subscriptions is so great that particularly the more energetic secretaries need to be reminded that commerce and charity have different ends and must be carried on by different principles.

### AN OLD CUSTOM ABANDONED.

THE old custom, which still persists, of requiring hospital in-patients to provide themselves with simple articles of food, is gradually falling into desuetude. It was hardly probable that it would long survive the introduction of contributions by patients toward the cost of their maintenance, and this reason, we notice, was expressly given for abandoning the custom at Bristol. There the committee of the General Hospital has decided to abolish the antiquated rule whereby patients had to bring their own "tea, sugar and butter." It is estimated that to provide these three articles will cost the Hospital £1,000 a year, though any patients who wish to furnish them will be allowed to do so. On the whole the disappearance of the custom is not to be regretted, and its abandonment is wise. Articles of diet should be up to the hospital standard, and this can hardly be when coming in small quantities from the most diverse and often impecunious homes.

### FAMILY INTEREST IN HOSPITALS.

SIR Leonard Lyle, whose knighthood, together with the recent visit of the King and Queen, has honoured Queen Mary's Hospital, West Ham, belongs to a family strong in hospital tradition. Both he and his father, Mr. Charles Lyle, have conferred innumerable benefits on Queen Mary's Hospital, of which the Margaret Lyle Maternity Wing is the latest only. All who are familiar with the development of this hospital during the past ten years know how good the work has been and how well its affairs have been guided. So long as the voluntary system can attract the interest of fathers and sons to the hospitals in their immediate neighbourhood, it has little to fear; and this kind of support is that which most deserves public honour.

